# The additional tibial stem extension is not mandatory for the stability of 5 mm metal block augmented tibial prosthesis construct in primary total knee arthroplasty: 5-year minimum follow-up results

**DOI:** 10.1186/s43019-023-00174-6

**Published:** 2023-02-01

**Authors:** Jae Joon Ryu, Yeong Hwan Kim, Choong Hyeok Choi

**Affiliations:** Department of Orthopaedic Surgery, College of Medicine, Hanyang University, Seoul, Republic of Korea

**Keywords:** Primary total knee arthroplasty, Bone defect, 5-mm metal augmentation, Stem extension

## Abstract

**Purpose:**

To determine whether additional stem extension for stability is necessary, we performed mid-term follow-up of patients who had been managed with 5-mm metal block augmentation for a tibial defect, where tibial prosthesis was fixed using bone cement without stem extension. Also, we evaluated clinical and radiologic results including survival rate of patients without stem extension.

**Methods:**

We retrospectively analyzed patients with tibial bone defect, had undergone primary total knee arthroplasty, and had been treated with 5-mm metal block augmentation without stem extension between March 2003 and September 2013. Among 74 patients (80 cases), 47 patients (52 cases) were followed up for at least 5 years.

**Results:**

Mean flexion contracture improved from 8.8° (0–40°) preoperatively to 0.4° (−5° to 15°) at final follow-up (*P* < 0.01), but there was no significant change in the mean angle of great flexion: 124.6° (75–150°) preoperatively and 126.2° (90–145°) at final follow-up (*P* = 0.488). Mean range of motion improved from 115.8° (35–150°) preoperatively to 125.5° (90–145°) at final follow-up (*P* < 0.01). Mean knee score improved from 38.7 points (0–66 points) preoperatively to 93.2 points (79–100 points) at final follow-up (*P* < 0.01), and mean functional score also improved from 50.4 points (10–70 points) preoperatively to 81.8 points (15–100 points) at final follow-up (*P* < 0.01). The mean postoperative Western Ontario and McMaster University osteoarthritis score was 19.5 points (0–66.0 points). The mean femorotibial angle was corrected from 9.0° varus (23.0° varus–6.3° valgus) preoperatively to 5.5° valgus (2.2° varus–11.1° valgus) at final follow-up (*P* < 0.01). There was no change in the mean β-angle, which was 90.7° (87.2–94.9°) immediately postoperative and 90.8° (87.2–94.9°) at final follow-up (*P* = 0.748) and in the mean δ-angle, which was 86.2° (81.3–90.0°) immediately postoperative and 87.2° (83.1–96.5°) at final follow-up (*P* = 0.272). Radiolucent lines (RLL) were observed in ten cases (26.3%), and the mean RLL scores at final follow-up were 0.34 points (0–3 points) in the anteroposterior view and 0.42 points (0–6 points) in the lateral view. Scores for the RLL were ≤ 4 points in 36 cases, 5–9 points in two cases. Revision surgery due to aseptic loosening (three cases) is rarely required, and the Kaplan–Meier survival rate at 10 postoperative years was 96.4%

**Conclusion:**

When performing 5-mm metal block augmentation for a proximal tibial defect, no additional tibial stem extension can be a good surgical option for the stability of tibial prosthetic construct and mid-term clinical and radiologic results.

**Level of evidence:**

IV.

## Introduction

Total knee arthroplasty (TKA) is a treatment method for advanced knee osteoarthritis (OA) that produces good clinical outcomes and high satisfaction [1–3]. As knee OA progresses, it leads to a varus deformity and a bone defect develops at the proximal tibial osteotomy site. Depending on its size, depth, and location, the bone defect can be managed by the methods of bone cement filling, bone cement and metal screw fixation, bone graft, or metal wedge or metal block augmentation[4–11]. Of these, metal block augmentation has the advantage of being a relatively easy method to treat the bone defect, but is unable to restore bony structures [12]. In using metal block augmentation to treat bone defects in the proximal tibia, stem extension is often recommended to enhance the stability of the tibial prosthesis[13, 14].

However, there are several problems in using additional stem extension. In the course of operation, tibial prosthesis could not be placed on the tibial cutting surface by extended stem sometimes. Moreover, the prosthesis costs related to stem extension cause financial burden. In addition, it can cause stem tip pain, which affects patient dissatisfaction [38]. Finally, when revision surgery is needed, large bone resection might be necessary, which may be followed by low bone density and technical problems such as fixation of revision implant [39, 40].

There are no clear guidelines on additional stem extension for the tibial component stability in primary total knee arthroplasty [15] The purpose of the study is to evaluate mid-term clinical and radiologic results of patients who had been managed with 5-mm metal block augmentation for a tibial defect, where tibial prosthesis was fixed using bone cement without stem extension. The authors hypothesize that they can show good clinical and radiologic results including survival rate without using stem extension.

## Materials and methods

### Patient treatment and evaluation

Among patients who had undergone primary TKA by a single surgeon at our hospital between March 2003 and September 2013, we retrospectively analyzed patients whose tibial bone defect had been treated with 5-mm metal block augmentation without stem extension. The study was approved by the consent of the System Review Committee of the Human Subject Research Ethics Committee.

Before the surgery, clinical data were assessed using Knee Society Scoring system (KSS) [16]. Preoperative alignment, deformity evaluation and surgical planning [17] were performed with both knees standing anteroposterior (AP), posteroanterior (PA), lateral, merchant view and whole lower extremity standing view.

During surgery, proximal tibial osteotomy was performed to generate a 4–7° posterior tibial slope in the sagittal plane, perpendicular to the anatomical axis of the tibia in the coronal plane. Thereafter, the medial tibial defect was measured, and for patients who could be treated with 5-mm metal block augmentation, a block cutting instrument was used to perform additional osteotomy of the medial tibia. A 5-mm metal block was fixed at the medial part of the tibial prosthesis using screws. The knee prostheses used for all the cases were posterior cruciate ligament-substituting prostheses (Scorpio, 42 cases or NexGen LPS, ten cases: Table 1). Metal-backed prostheses were used in all cases, and the length of the basic stem connected to the tibial plate was 30–40 mm. Additional stem extension was not performed. The whole prosthesis, including under-surface of tibial plate, basic stem, and under-surface of 5-mm metal block, was fixed to the tibia with fully covered cementation.

**Table 1:** Characteristics of patients

	Data (n* = 52)
F/U period	106.9 (60.6–185.3)
Age	65.0 (40–79)
Sex (M:F)	5: 47
OA	44
RA	8
TKA prosthesis	All PS types
Scorpio	42
NexGen	10

Continuous variables as mean (range)

*F/U* follow up, *OA* osteoarthritis, *RA* rheumatoid arthritis, *TKA* total knee arthroplasty

*Number of cases

After surgery we took immediate postoperative knee AP and lateral view [18] for evaluating femorotibial alignment and tibial prosthesis position. In addition, these data were used as a reference point for comparison with the image at the time of last follow-up.

Radiologic and clinical evaluation was performed on regular outpatient visits (3 months, 6 months, 1 year, and annually) after surgery, and additional tests were performed if patients had symptoms. The clinical outcomes were assessed using the Knee Society Scoring system (KSS) and the Western Ontario and McMaster Universities (WOMAC) osteoarthritis index [19]. The American Knee Society radiological assessment method [20] was used for radiologic evaluation. The femorotibial alignment and tibial prosthesis position (β and δ angle) were checked with both knees standing AP, lateral, and whole lower extremity standing view and radiolucent line scores were evaluated with tibial prosthesis focused anteroposterior (AP) and lateral view knee fluoroscopy images.

### Study population

**Fig. 1:**
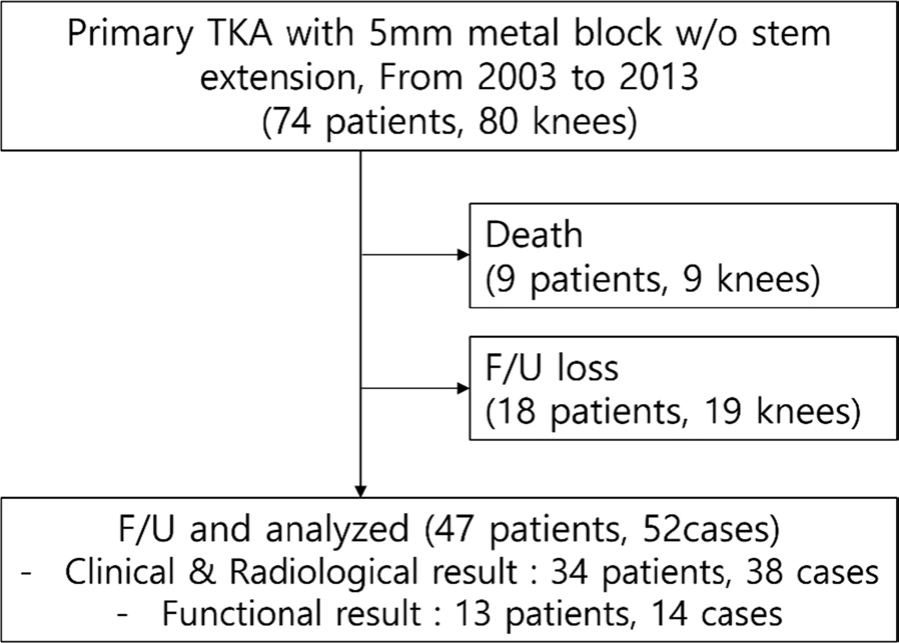
Flowchart summarizing the follow-up process

Among 74 patients (80 cases) who underwent primary TKA using a 5-mm metal block without stem extension for their tibial defect, 9 patients (9 cases) died, 18 patients (19 cases) were lost to follow-up, and the remaining 47 patients (52 cases) were followed up for at least 5 years. For 13 of these patients (14 cases), the KSS function score and revision surgery status were confirmed by telephone contacts, while the other 34 patients (38 cases) underwent clinical and radiological assessment at final follow-up (Fig. 1). The mean age at the time of surgery was 65.0 years (40–79 years). There were 43 female patients (47 cases) and 4 male patients (5 cases), and 39 patients (44 cases) were diagnosed with OA and 8 patients (8 cases) with rheumatoid arthritis (Table 1).

### Statistical analysis

Paired *t*-test was used for statistical comparisons of pre- and postoperative clinical and radiological outcomes. Kaplan–Meier survival analysis was used for the progress of TKA using a 5-mm metal block without stem extension, and the end point of event was defined as the time of performing the revisional surgery due to the problem of the tibial component. Statistical significance was set at *P*-value < 0.05. All statistical analyses were performed using IBM-SPSS v.26 software (SPSS Inc., Chicago, USA).

## Results

The mean follow-up duration was 8 years, 11 months (60.6–185.3 months). All clinical and radiological results showed significant improvement between preoperative and final follow-up evaluation. The comparisons between postoperative radiological parameter and final follow-up evaluation for implant stability showed no significant difference.

Mean flexion contracture improved from 8.8° (0–40°) preoperatively to 0.4° (−5° to 15°) at final follow-up (*P* < 0.01), but there was no significant change in the mean angle of greatest flexion (AGF): 124.6° (75–150°) preoperatively and 126.2° (90–145°) at final follow-up (*P* = 0.488). Mean joint range of motion (ROM) improved from 115.8° (35–150°) preoperatively to 125.5° (90–145°) at final follow-up (*P* < 0.01; Table 2). Among patients with at least 5 years of postoperative follow-up, mean knee score improved from 38.7 points (0–66 points) preoperatively to 93.2 points (79–100 points) at final follow-up (*P* < 0.01), and mean functional score also improved from 50.4 points (10–70 points) preoperatively to 81.8 points (15–100 points) at final follow-up (*P* < 0.01). The mean postoperative WOMAC OA score was 19.5 points (0–66.0 points; Table 3).

**Table 2:** Comparison between preoperative and final follow-up range of movement

	Preoperative	Final F/U	95% confidence interval	*P*-value
AGE	8.8 (0–40)	0.4 (−5 to 15)	−11.67 to 5.17	< 0.01
AGF	124.6 (75–150)	126.2 (90–145)	−2.99 to 6.15	0.488
ROM	115.8 (35–150)	125.5 (90–145)	3.32–16.15	< 0.01

Continuous variables as mean (range)

*AGE* angle of greatest extension, *AGF* angle of greatest flexion, *ROM* range of motion

**Table 3:** Comparison between preoperative and final follow-up KSS and final follow-up WOMAC OA index

	Preoperative	Final F/U	95% confidence interval	*P*-value
Knee score (*n* = 38)	38.7 (0–66)	93.2 (79–100)	47.63–0.47	< 0.01
Function score (*n* = 52)*	50.4 (10–70)	81.8 (15–100)	22.99–39.90	< 0.01
WOMAC OA index (*n* = 19)		19.5 (0–66)		

Continuous variables as mean (range)

*Including functional follow-up group

For radiological outcomes of the tibial prostheses augmented with a 5-mm block, the mean femorotibial angle was corrected from 9.0° varus (23.0° varus–6.3° valgus) preoperatively to 5.5° valgus (2.2° varus–11.1° valgus) at final follow-up. There was no change in the mean β-angle, which was 90.7° (87.2–94.9°) immediately postoperative and 90.8° (87.2–94.9°) at final follow-up (*P* = 0.748) and in the mean δ-angle, which was 86.2° (81.3–90.0°) immediately postoperative and 87.2° (83.1–96.5°) at final follow-up (*P* = 0.272; Table 4).

**Table 4:** Comparison between preoperative, postoperative, and final follow-up radiological alignment and tibial prosthesis position

	Preoperative	Postoperative	Final F/U	95% CI	*P*-value
TFA	−9.0 (−23.0 to 6.3)		5.5 (−2.2 to 11.1)	12.78–16.34	< 0.01
β-Angle		90.7 (87.2–94.9)	90.8 (87.2–94.9)	−0.42 to 0.58	0.748
δ-Angle		86.2 (81.3–90.0)	87.2 (83.1–96.5)	−0.28 to −0.96	0.272

Continuous variables as mean (range)

*TFA* tibiofemoral angle, *CI* confidence interval

For the patients who had been evaluated with knee fluoroscopy (38 cases), radiolucent lines around the tibial prosthesis were observed in 10 cases (26.3%), and the mean radiolucent line scores at final follow-up were 0.34 points (0–3 points) in the AP view and 0.42 points (0–6 points) in the lateral view. None of the patients showed findings that suggest instability. Scores for the radiolucent line were ≤ 4 points in 36 cases, 5–9 points in 2 cases, and ≥ 10 points in none of the cases. The areas where radiolucent lines were observed were the medial part of AP views in six cases, the lateral part of AP views in two cases, the anterior part of lateral views in five cases, and the posterior part of lateral views in one case. No radiolucent lines were observed around the stem of the tibial prosthesis (Table 5). Radiolucent lines were observed after 1 postoperative year in eight cases and after 4 postoperative years in two cases; apart from one case with rotational displacement of the tibial component where the radiolucent line score increased by 4 points after 8 postoperative years, the radiolucent lines did not progress in these cases. One case showed a high radiolucent line score of 9 points immediately after surgery and was followed up regularly, but the patient continually showed excellent clinical outcomes. Apart from this case, no case showed findings of aseptic loosening or osteolysis; therefore, no case required revision arthroplasty.

**Table 5:** Radiolucent line score and distribution in clinical and radiological follow-up cases (38 cases)

Total RLL score	0	1–4	5–9	≥ 10
	28 cases	8 cases	2 cases	0 cases

*RLL* radiolucent line

**Fig. 2:**
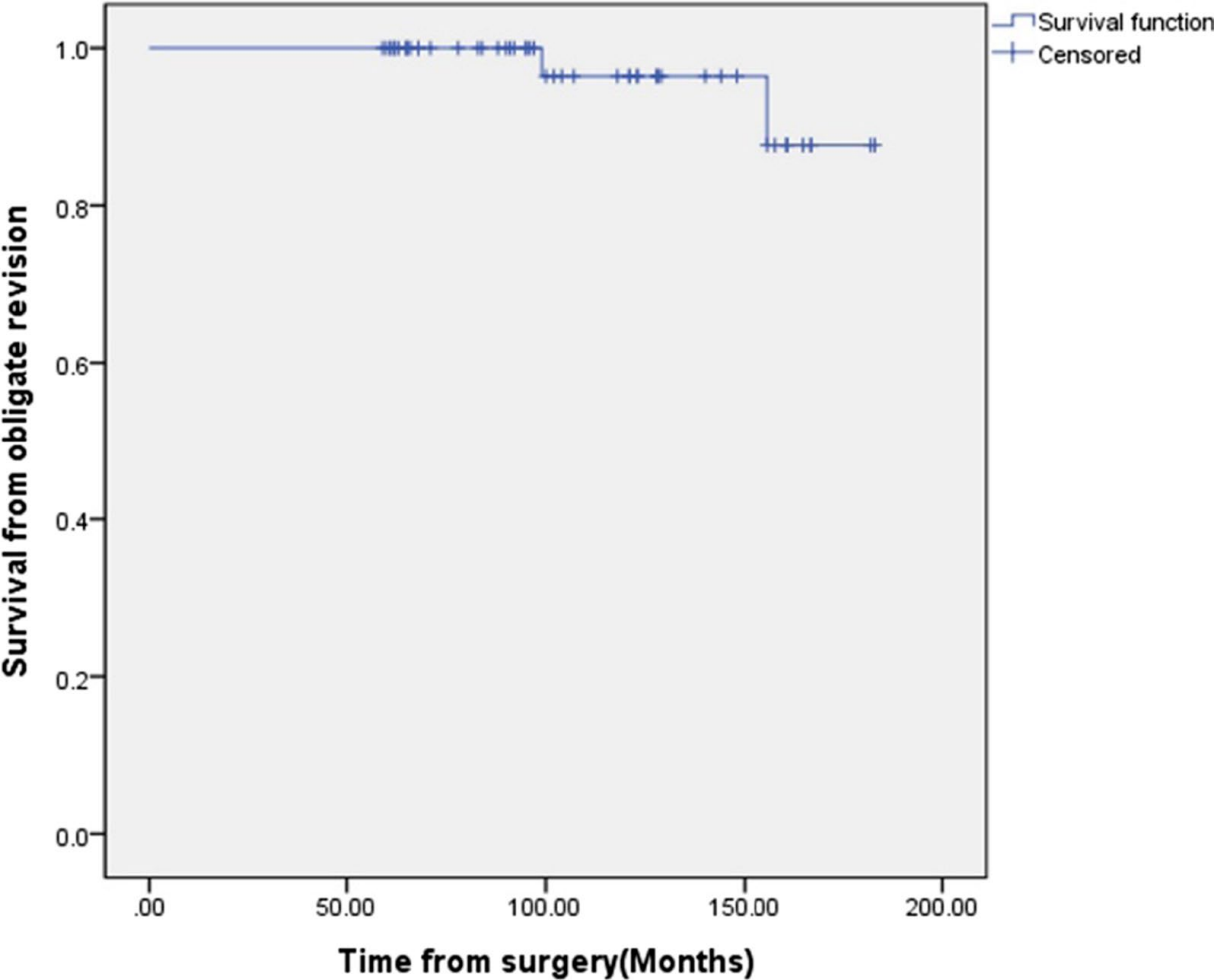
Kaplan–Meier curve analysis of primary total knee arthroplasty with a 5-mm metal block augmentation without stem extension

Finally, revision surgery due to aseptic loosening (three cases) is rarely required, and the Kaplan–Meier survival rate at 10 postoperative years was 96.4% (Fig. 2).

## Discussion

The most important finding of this study is that 5-mm metal block augmentation with no additional tibial stem extension brought about good clinical and radiological results with no stability problems for the proximal tibial defect management during primary total knee arthroplasty in at least 5 years mid-term follow-up period.

In advanced OA requiring TKA, varus deformity is often observed, and in such cases, bone defects of the medial tibia are very common[21]. Depending on the location, size, and depth of the bone defect, primary TKA methods such as lateralization of the tibial prosthesis, cement filling [10], bone grafting [4, 8], or metal block augmentation are used to treat the medial tibial bone defect, and metal augmentation is one of the easiest of these methods [12]. Because metal block augmentation involves removal of the defective site, the bone mass cannot be restored; on the other hand, potential problems with bone graft such as incorporation failure or collapse, are not expected [22]. Lee and Choi [12] reported favorable outcomes at a 5-year follow-up, and Tsukada et al. [23] reported that clinical results of total knee arthroplasty with metal augmentation showed no differences compared with those without bone defects.

There are some doubts whether the basic tibial prostheses designed for the primary TKA might have stability problems caused by partial modification with metal block attachment. Therefore, additional tibial stem extension has been suggested for the increasing stability when metal block augmentation is attached to the tibial component. Stem extension is expected to enhance the stability [12, 24, 25] because it can provide shear force resistance [26], reduce tibial lift-off [27], and mitigate micromotion [28]. The length of stem extension has been reported to influence stability [29–32]; hence, when performing metal block augmentation of a tibial prosthesis, stem extension is recommended [13, 14].

However, there have also been reports that, when an eccentric load is applied in stem extension, micromotion increases; therefore, stem extension does not increase initial stability in TKA [31, 33]. As loading on the proximal tibia is reduced, the bone density also decreases [26] due to the stress shielding effect occurs along the stem. This in turn increases the risk of subsidence, loosening, and periprosthetic fracture [34]. Meanwhile, when stress is focused on the stem tip, it can lead to stem tip pain [34, 35]. Additional problems that need to be considered are the heightened risk of fat embolism during intramedullary manipulation and implant placement for an intramedullary stem extension, increased prosthesis costs due to the metal block and stem, and clinically, potential loss of bone quality and difficulties of stem removal in a future revision surgery.

In a biomechanical study that assessed metal block augmentation and stem extension for proximal tibial defects, performing a stem extension was reported to produce favorable outcomes compared with using only the metal block [15]. However, that study had certain limitations: it included only relatively large bone defects, it used a 10-mm metal block augmentation, and the stability results were assessed at time zero state and applied on the synthetic bone rather than real bony structure. When using a cement fixation method for tibial prosthesis, cementation of the tibial cutting surface only and cementation that also includes the tibial stem are known to show differences in stability. Cawley et al. [36] pointed out that, even though full cementation of the surface and stem provided excellent initial stability, there was a risk of proximal tibial bone resorption with time because there was a lot of stress shielding. Therefore, the stability at time zero is not the ultimate measurement in typical patients, and with time, it is also important to monitor for aseptic loosening indicated by bony absorption in the proximal tibia.

From the results of this study, there were positive outcomes in clinical parameter including flexion contracture, ROM and KSS at final follow-up among primary TKA patients who underwent 5-mm block augmentation without stem extension and were available for at least 5 years of follow-up. And there was no change in β-angle or δ-angle after a mean of 9 years of follow-up compared to angles measured immediately after surgery. Radiolucent line scores around the tibial implant, investigated by fluoroscopy, were mostly ≤ 4 points at final follow-up, indicating sustained stability.

Among the 47 patients (52 cases) who were followed up for at least 5 years, there were 3 cases related to revision surgery; 1 case in which revision surgery was performed, 1 case in which revision surgery was advised, and 1 case in which continuous observation was advised. In the case where revision surgery was performed, there were no clinical and radiological signs of loosening during 4 postoperative years. However, at 13 postoperative years, the patient underwent revision surgery for aseptic loosening at the other hospital. Also, in the case where revision surgery was advised, there were no signs of loosening for up to 7 postoperative years. But at 8 postoperative years, revision surgery was advised due to rotational displacement of the tibial component.

In this study, revision surgery was recommended in 3 cases and the Kaplan–Meier survival rate for 10 years after surgery was 96.4%, which is not much different from 97%, the 10-year survival rate of primary TKA reported by Nugent et al. [37], and 95.3% reported by Khaw et al. [41]. However, since comparative analysis of survival rates has not been conducted, studies using statistical techniques such as log-rank test should be conducted to see if there is a difference in survival rates according to the implementation of stem extension.

**Fig. 3:**
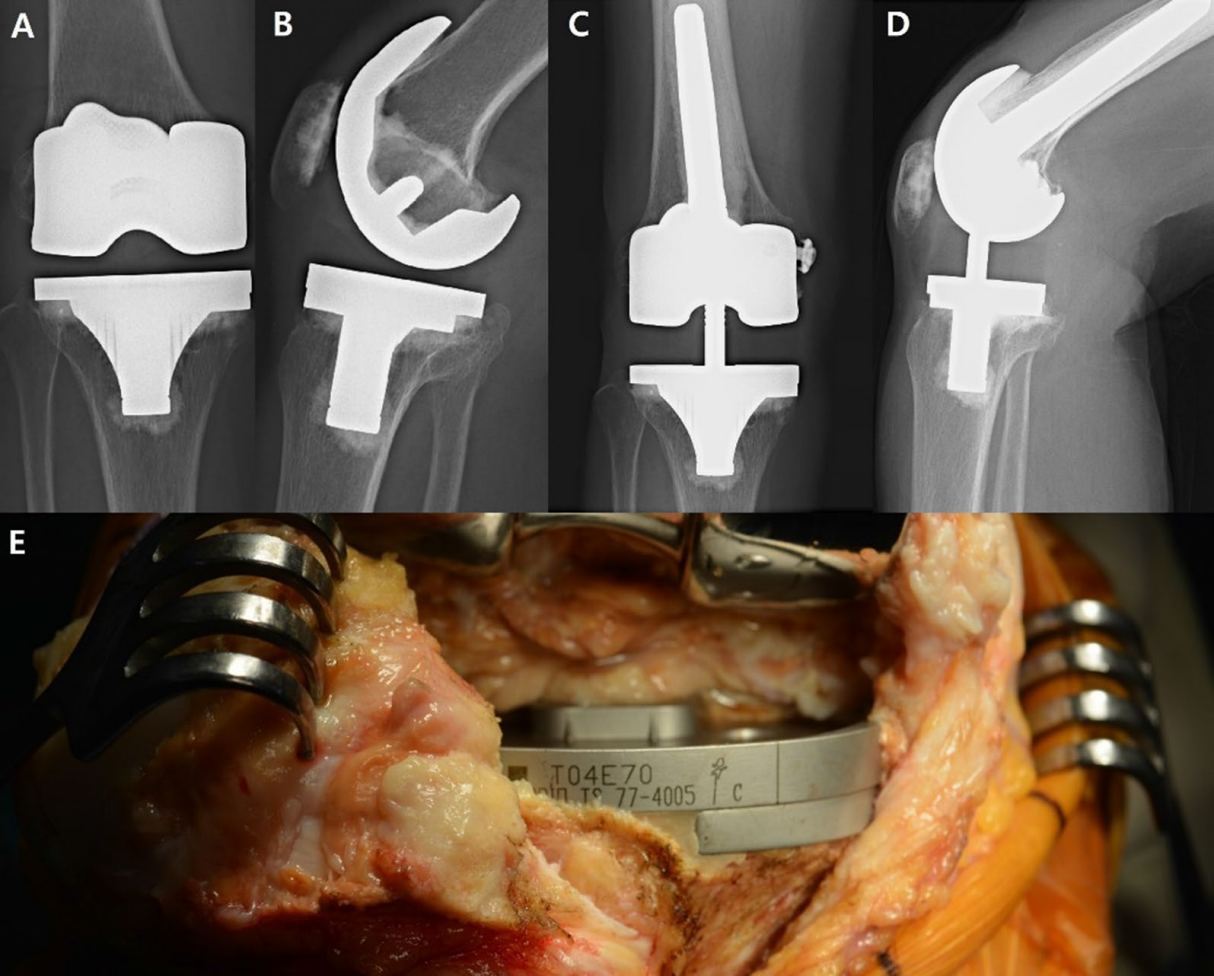
**A**, **B** Preoperative fluoroscopy on right knee (post-operative day (POD) POD#11.5 years); **C**, **D** postoperative fluoroscopy on right knee (POD#12.8 years); **E** intraoperative clinical photo on revision TKA on right knee (POD#11.5 years)

Among the cases, one patient is under the continuous monitoring for 9 points of the radiolucent line score, noticed immediate postoperative state, but there was still no interval changes in the radiolucent line scores till 90 postoperative months and the clinical outcomes were excellent (knee score 100, function score 90). So this patient is still followed up very closely. The other patient who underwent 5-mm metal block augmentation without stem extension in her primary TKA revised only the femoral component at 11.5 postoperative years. However, the tibial component showed good stability (Fig. 3) in the revision procedure. After the femoral component revision surgery, the patient continued to show excellent knee/function scores and no radiolucent line around the tibial prosthesis. Therefore, we concluded that, in primary TKA patients, a 5-mm metal block augmentation of tibial bone defects without stem extension can provide stability in 5-year postoperative outcomes.

This study was conducted to answer the question of the necessity of stem extension for metal block augmentation of medial defects on the basis of clinical outcomes after at least 5 years of follow-up. We were able to demonstrate that, in mid-term follow-up of at least 5 years and an average of 9 years, no additional stem extension is required to the 30–40 mm basic length tibial stem plate for the stability with fully cemented tibial component when performing 5-mm metal block augmentation in primary TKA.

However, this study also had several limitations. First, since this study is a case series for patients who underwent 5-mm metal block augmentation without stem extension, it was possible to confirm improvement in clinical and radiological results compared with before surgery. However, it reveals the limitations of not performing non-inferiority test because there is no comparison group with stem extension.

Second, we only evaluate no additional tibial stem extension cases for the focal proximal tibial defect, which is managed with 5-mm metal block augmentation. So, our results may not be applied to the situation of generalized poor bone quality on the proximal tibia.

Third, there were relatively many patients who could not receive clinical and radiation follow-up measures due to the long data collection period, especially elderly patients who died or had other accompanying diseases. This also has a limitation in that selection bias may appear because there are patients who have been eliminated during long period.

## Conclusion

When performing TKA, 5-mm metal block augmentation can be used to deal with uncontained bone defects less than 5-mm depth. If the tibial component with 30–40 mm basic stem was fixed through full cementation, the additional tibial stem extension was not mandatory for the stability of tibial prosthesis and mid-term clinical and radiologic results. Therefore, when performing 5-mm metal block augmentation for a proximal tibial defect, no additional tibial stem extension can be a good surgical option.

## Abbreviations

TKA: Total knee arthroplasty
OA: Osteoarthritis
KSS: Knee Society Scoring system
WOMAC: Western Ontario and McMaster Universities
AP: Anteroposterior
AGF: Angle of greatest flexion
ROM: Range of motion
RLL: Radiolucent line
